# Divergent roles of H3K18 lactylation in human diseases: metabolic-epigenetic circuits and translational challenges

**DOI:** 10.3389/fgene.2026.1950620

**Published:** 2026-10-26

**Authors:** Ting Wu, Yali Zhang, Yifei Liu

**Affiliations:** Department of Pathology, Affiliated Hospital of Nantong University, Nantong, China

**Keywords:** epigenetic regulation, H3K18 lactylation, histone lactylation, inflammation, lactate metabolism, molecular medicine, tumor microenvironment

## Abstract

Histone H3 lysine 18 lactylation (H3K18la) links fluctuations in lactate metabolism with chromatin-dependent transcription. Promoter- and enhancer-level associations suggest that H3K18la can report local metabolic-chromatin coupling, but evidence for modification-specific causality remains uneven because the known writers and erasers are promiscuous, dedicated readers are poorly defined, and broad acyltransferase inhibitors perturb competing histone and non-histone marks. Across tumor, inflammatory, metabolic, cardiovascular, and degenerative-disease models, H3K18la participates in oncogenic, immunoregulatory, fibrotic, or reparative programs according to cell lineage, chromatin state, metabolic exposure, and disease stage. We synthesize these observations using a testable framework in which lactate and lactyl-CoA provide metabolic permission, whereas transcription factors, chromatin accessibility, histone chaperones, and candidate protein or RNA scaffolds provide locus selection; downstream readers or conventional transcriptional effectors then shape the biological output. Direct long non-coding RNA (lncRNA)-guided deposition and a universally validated H3K18la reader remain unproven. Human cohort data support exploratory, disease-specific associations, but assay heterogeneity and the lack of prospective external validation preclude current clinical use. Progress will require histone H3 lysine 18 acetylation (H3K18ac)-aware, inhibitor-independent causal tests, structural validation of readers, integrated chromatin-RNA profiling, and prospective multicenter cohorts.

## 1 Introduction

The nucleosome is the fundamental structural unit of eukaryotic chromatin. It consists of an octamer formed by the core histones H2A, H2B, H3, and H4, around which approximately 147 bp of DNA is wrapped; linker histone H1 further stabilizes higher-order chromatin architecture and promotes chromatin compaction (Ozer et al., 2015). Histone post-translational modifications (PTMs) are central epigenetic mechanisms that regulate chromatin conformation, DNA accessibility, and transcription, and they are widely implied in metabolic reprogramming, immune homeostasis, and pathological tissue remodeling (Rothbart and Strahl, 2014). Among the core histones, histone H3 contains a particularly dense set of functionally diverse modification sites. Its flexible N-terminal tail projects outside the nucleosome and provides an accessible platform for receiving and integrating upstream signals. Lysine 18 on histone H3 (H3K18), located in this exposed N-terminal region, is highly conserved and structurally accessible (Figure 1). At lysine 18 (H3K18), reported modifications include methylation (Cheeseman et al., 2021), ubiquitination (Liu et al., 2025), crotonylation (Weng et al., 2026), and acetylation (Zeng et al., 2025). Together, these studies establish H3K18 as a site at which chemically distinct modifications can support context-dependent regulatory programs.

**FIGURE 1:**
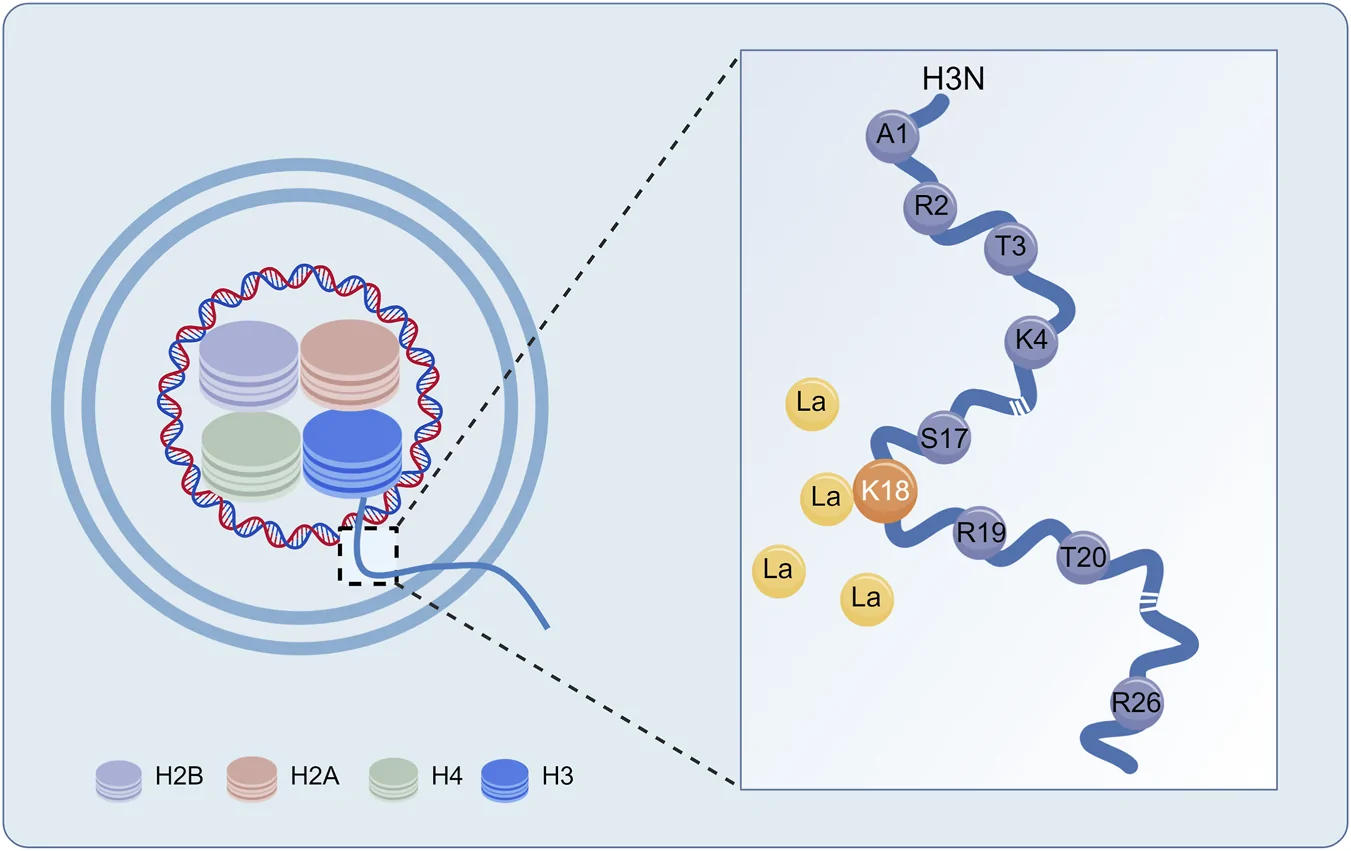
Schematic diagram of lactylation modification at K18 on the N-terminal tail of histone H3. The left side shows nucleosomes, the fundamental repeating unit of chromatin, with four core histones distinguished by distinct color blocks. The right panel magnifies the flexible N-terminal sequence of H3 to visualize lactate moieties (La) conjugated to lysine 18.

Histone H3 lysine 18 acetylation (H3K18ac), the best-characterized acyl mark at this residue, provides an important comparator for histone H3 lysine 18 lactylation (H3K18la). H3K18ac is deposited mainly by the lysine acetyltransferases p300 and cyclic AMP response element-binding protein (CBP), is linked to open chromatin and active transcription, and has been evaluated by immunohistochemistry as a prognostic biomarker in several human cancers (Haasa et al., 2019). Its associations are tumor dependent: low global H3K18ac was associated with poorer outcome in some lung and kidney cohorts (Seligson et al., 2009), whereas high H3K18ac has been associated with adverse outcome in pancreatic cancer (Juliano et al., 2016) and hepatocellular carcinoma (Lee et al., 2016). These discordant directions, together with the use of H3K18ac as the corresponding-lysine comparator in lactylation studies, underscore the need to measure both marks and to avoid interpreting H3K18la without its acetylation context.

Histone lactylation was first reported in 2019 by the group of Yingming Zhao at the University of Chicago as a previously unrecognized PTM (Zhang et al., 2019). In this modification, a lactyl group derived from the cellular metabolite lactate is enzymatically attached to lysine residues on histones. The discovery reframed lactate from a presumed metabolic by-product into a signaling metabolite that can directly influence gene transcription through chromatin modification. H3K18la, one of the best-studied sites in the histone lactylation family, refers chemically to the addition of a lactyl group to the epsilon-amino group of H3K18. At the genomic level, H3K18la is preferentially enriched by promoters and enhancers of actively transcribed genes and is generally associated with transcriptional activation (Zhang et al., 2019).

Although the biological functions of H3K18la are beginning to emerge, its fine regulatory architecture remains incompletely resolved. Current studies implicate that reversible H3K18la dynamics depend mainly on broad-spectrum acyltransferases, such as p300/CBP in H3K18la deposition (Zhang et al., 2019) and class I histone deacetylases (HDACs), including histone deacetylase 3 (HDAC3), in removal (Moreno-Yruela et al., 2022). However, truly site-specific “writers” and “erasers” have not yet been clearly defined (Haasa et al., 2019; Seligson et al., 2009). In the glioblastoma microenvironment, for example, lactylated histones can interact with the heterochromatin component chromobox protein homolog 3 (CBX3); although CBX3 itself has no direct lactyltransferase activity, but it recruits p300 and shifts its substrate preference toward lactyl-CoA, thereby driving an immunosuppressive transcriptional program (Wang et al., 2024a). ACSS2 has also been identified as a lactyl-CoA synthetase that converts lactate into lactyl-CoA and assembles with KAT2A; within this complex, KAT2A functions as a lactyltransferase that catalyzes histone H3 lactylation, upregulates Wnt/β-catenin, NF-κB, and programmed death-ligand 1 (PD-L1) signaling, and promotes brain tumor growth and immune escape (Zhu et al., 2025). These findings emphasize the complexity of H3K18la regulation and explain why the absence of specific regulatory enzymes remains a major barrier to precise mechanistic interpretation. Likewise, the upstream signaling cascades and downstream target-gene networks of H3K18la are still only partially mapped. In pancreatic cancer, for instance, H3K18la levels correlate positively with serum lactate, carbohydrate antigen 19–9 (CA19-9), and carcinoembryonic antigen (CEA), but how upstream metabolic signals are sensed and converted into changes in H3K18la abundance remains to be clarified (Hou et al., 2025).

Despite these gaps, accumulating evidence indicates that H3K18la is dysregulated in diverse pathological settings and contributes to disease progression by modulating cell proliferation, immune responses, and metabolic reprogramming. In colon cancer, H3K18la promotes cancer-stem-cell enrichment by upregulating stemness-related genes (Zhang Q. et al., 2025). In hepatocellular carcinoma (HCC), it activates M2 macrophage markers, including CD206, ARG1, and IL10, promotes tumor-associated macrophage polarization, suppresses CD8^+^ T-cell infiltration and cytotoxicity, and establishes an immunosuppressive microenvironment (Cai et al., 2024). In lung cancer, H3K18la has been associated with POM121 upregulation *MYC* nuclear translocation and binds the *CD274* promoter, and increased PD-L1 expression; the reported studies link this axis to immune escape (Zhang et al., 2024a). In inflammatory and immune-related diseases, H3K18la is increased in peripheral blood monocytes from patients with septic shock and is associated with disease severity, cytokine-storm intensity, and arginase 1 expression, supporting its potential as a biomarker for sepsis assessment (Chu et al., 2022). In rheumatoid arthritis (RA), lactate accumulation in synovial fluid increases H3K18la and inflammatory cytokine release, whereas Parishin E reduces H3K18la levels by enhancing SIRT6 activity and alleviating joint inflammation and tissue injury (Xu et al., 2026). During chronic kidney disease, PKM2-driven lactate accumulation upregulates TGF-β1 through H3K18la, promotes macrophage-to-myofibroblast transition, and accelerates renal fibrosis (Xiang et al., 2025). In degenerative disease, H3K18la can activate osteogenic gene networks and promote bone marrow mesenchymal stem cell (BMSC) differentiation toward osteoblasts, thereby partially attenuating osteoporosis progression (Wu et al., 2023). Collectively, these observations support a substantial role for H3K18la in pathological remodeling, while its clinical utility as a diagnostic marker or therapeutic target still requires rigorous validation.

Taken together, current studies place H3K18la at the interface of cellular metabolism and transcriptional regulation (Figure 2). The available mechanistic evidence spans tumor-microenvironment remodeling, inflammatory signaling, metabolic injury, and degenerative disease, but the responsible enzyme complexes and causal downstream pathways remain incompletely resolved. Accordingly, this review compares disease-specific mechanisms with the limitations that currently restrict translation, providing a basis for judging where H3K18la is mechanistically informative and where further validation is required.

**FIGURE 2:**
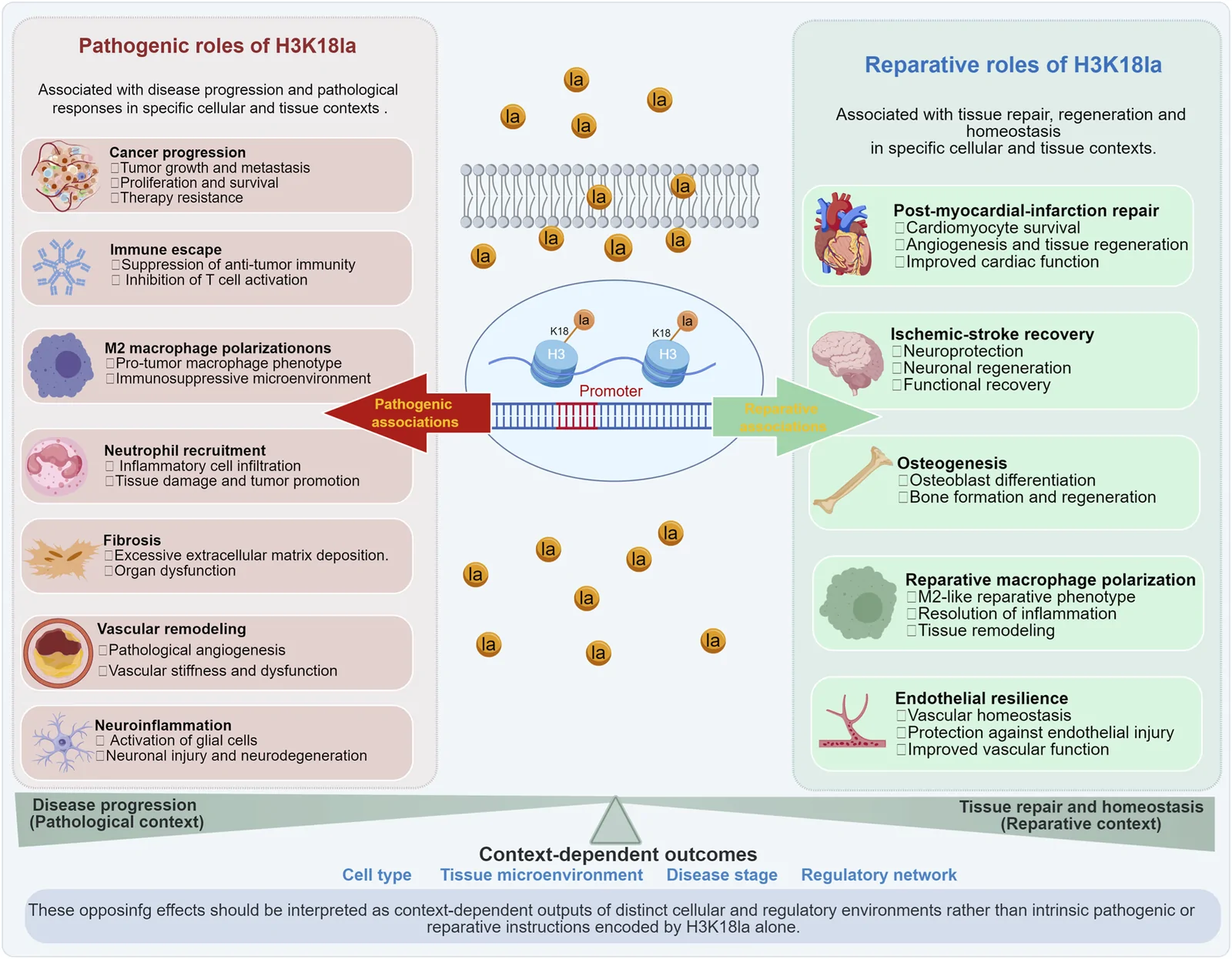
Context-dependent pathogenic and protective/reparative roles of H3K18 lactylation in human diseases. H3K18 lactylation (H3K18la) at promoter regions is associated with divergent biological outcomes across tissues and disease states. Its pathogenic associations include cancer progression, immune escape, M2 macrophage polarization, neutrophil recruitment, fibrosis, vascular remodeling, and neuroinflammation. Conversely, H3K18la is also associated with post-myocardial-infarction repair, ischemic-stroke recovery, osteogenesis, reparative macrophage polarization, and endothelial resilience. These opposing effects should be interpreted as context-dependent outputs of distinct cellular and regulatory environments rather than intrinsic pathogenic or reparative instructions encoded by H3K18la alone.

## 2 Regulatory mechanisms of H3K18la

### 2.1 Sources and regulation of metabolic precursors

Lactate flux and lactyl-CoA availability contribute to H3K18la deposition, but the quantitative relationship depends on compartmentalization, enzyme complexes, and competing acyl-CoA species (Zhang et al., 2019). The concentration of free intracellular lactate is therefore considered a rate-limiting determinant of H3K18la abundance. Lactate levels are shaped by the balance between lactate dehydrogenase isoenzymes (LDHA/LDHB) and the expression of monocarboxylate transporters (MCT1/MCT4) (Doherty and Cleveland, 2013). Under hypoxia or high-glucose conditions, cells preferentially rely on glycolysis, leading to lactate accumulation, increased substrate availability, and elevated H3K18la (Zhang et al., 2019; Gaur et al., 2026). Across disease settings, this relationship has been described as a metabolism-epigenetics feedback circuit (Zhang L. et al., 2025). In papillary thyroid carcinoma, tumor-derived lactate promotes H3K18la and enhances transcriptional activation of signal transducer and activator of transcription 1 (STAT1); activated STAT1 then upregulates LDHA transcription, increases lactate biosynthesis, and sustains a positive feedback loop that supports malignant progression (Zhou Z. et al., 2025). Exogenous lactate experiments further show that extracellular lactate can increase global histone lactylation in a dose-dependent manner, with H3K18la showing a particularly marked response (Moreno-Yruela et al., 2022). These findings indicate that dynamic changes in cellular lactate flux are a central driver of H3K18la variation.

### 2.2 Enzymatic regulatory network

The enzymatic network governing H3K18la remains at an early stage of characterization, and no universally validated site-specific writer or eraser has been established. p300/CBP was implicated in histone lactylation (Zhang et al., 2019), whereas ACSS2-derived lactyl-CoA enables KAT2A/GCN5-mediated histone H3 lactylation (Zhu et al., 2025). Structural and biochemical studies show that p300 accepts multiple acyl-CoA cofactors; acetyl-CoA is used most efficiently, but propionyl-, crotonyl-, and butyryl-CoA can also occupy the catalytic tunnel, with activity generally decreasing as acyl-chain size increases (Kaczmarska et al., 2017). p300 also catalyzes histone 2-hydroxyisobutyrylation (Huang et al., 2018) and crotonylation (Sabari et al., 2015). Thus, p300/CBP perturbation changes a broad acylation landscape rather than H3K18la alone. Class I HDACs and SIRT2 exhibit delactylase activity but have not been shown to selectively remove H3K18la (Moreno-Yruela et al., 2022; Zu et al., 2022). C646 is not H3K18la-selective (Chen et al., 2022), whereas A-485 is p300/CBP enzyme-selective but modification-nonselective (Lasko et al., 2017). A change in H3K18la after shared-enzyme perturbation cannot by itself establish H3K18la-specific causality. Strong causal studies should combine targeted H3K18la quantification with H3K18ac and pan-acylation controls, orthogonal metabolic perturbation, and locus-specific rescue or epigenome editing.

### 2.3 Upstream and downstream targets and signaling pathways

As an epigenetic regulator, H3K18la exerts many of its biological effects by altering chromatin state and activating downstream transcriptional programs. Integrative chromatin immunoprecipitation sequencing (ChIP-seq) and RNA sequencing (RNA-seq) analyses have identified several core genes directly associated with H3K18la, including *MYC*, *HIF1A*, *IL6*, and *VEGFA* (Peng and Du, 2025). These targets provide a molecular basis for cell proliferation, metabolic reprogramming, and immune microenvironment remodeling. A parsimonious model for locus specificity separates metabolic permission from local recruitment. Lactate flux, nuclear lactyl-CoA supply, and competition among acyl-CoA species determine whether lactylation is chemically possible; cell-lineage transcription factors, pre-existing chromatin accessibility, histone chaperones, and protein or RNA scaffolds then concentrate promiscuous acyltransferases at selected loci. CBX3 recruitment of EP300 in glioblastoma (Wang S. et al., 2024), ACSS2-KAT2A complex formation (Zhu et al., 2025), and ASF1A-dependent p300 activity at the SNAI1 locus (Dong et al., 2024) provide examples of such contextual scaffolding. Long non-coding RNAs (lncRNAs) are established modular scaffolds for histone-modifying complexes (Tsai et al., 2010) and are therefore plausible additional targeting factors. In the current H3K18la literature, the lncRNA H19-glycolysis-histone-lactylation loop in gastric cancer (Tsukihara et al., 2025) and the circular RNA circXRN2-Hippo-H3K18la pathway in bladder cancer (Xie et al., 2023) show that non-coding RNAs can regulate the abundance or consequences of lactylation. However, direct RNA-mediated tethering of p300/CBP or KAT2A to a defined H3K18la target promoter has not yet been demonstrated. RNA pulldown, cross-linking and immunoprecipitation (CLIP)-based interaction mapping, chromatin isolation by RNA purification sequencing (ChIRP-seq), and loss-of-function studies coupled to H3K18la ChIP-seq are needed to test this mechanism. The effector step is equally unresolved. In hepatocellular carcinoma, BRD9 was reported to recognize H3K18la (Wei et al., 2026), though there remains no widely accepted reader protein specific to this modification. Key structural studies on other acyl-lysine modifications illustrate how reader selectivity arises from binding-pocket geometry, hydrogen-bond formation and aromatic interactions. A limited subset of human bromodomains can bind butyryl- or crotonyl-lysine residues (Flynn et al., 2015). The YEATS domains, by comparison, favour crotonyl-lysine *via* aromatic-sandwich interactions (Li et al., 2016; Zhang et al., 2016). These observations warn against inferring H3K18la-reading activity merely from known acetyl-lysine-binding domains.Without supporting evidence from biochemical affinity assays, structural analysis, genomic colocalization data and reader-dependent rescue experiments, it is more reasonable to consider H3K18la as a marker of transcription-permissive chromatin. It likely functions together with locally recruited transcription factors and chromatin remodelers, instead of defining promoter identity on its own.

## 3 Key roles of H3K18la in tumor progression

### 3.1 Promotion of tumor cell growth and cell-cycle progression

Studies using human tissue samples offer preliminary, disease-specific evidence linking H3K18la to patient prognosis. In pancreatic ductal adenocarcinoma (PDAC), elevated H3K18la levels in tumor tissue correlate with reduced patient survival (Li F. et al., 2024). In gastric cancer, a molecular profile featuring high H3K18la alongside low SIRT1 expression was associated with worse clinical outcomes (Tsukihara et al., 2025). In hepatocellular carcinoma (HCC), H3K18la participates in a prognostically important regulatory network centered on SRSF10 (Cai et al., 2024); in non-small cell lung cancer (NSCLC), this histone modification acts within a KRT19-centered circuit (Zhang C. et al., 2026). Even so, the survival analyses in those studies focused mainly on SRSF10 and KRT19 and did not independently confirm H3K18la as an individual prognostic biomarker.

These retrospective correlative findings cannot be equated with causal mechanistic data from inhibitor experiments. C646 lacks selectivity toward H3K18la (Chen et al., 2022), and blocking p300/CBP perturbs wide-ranging protein acylation events (Kaczmarska et al., 2017; Huang et al., 2018; Sabari et al., 2015; Lasko et al., 2017). Neither line of evidence proves H3K18la itself drives disease phenotypes, nor supports its use as a validated biomarker. Published cohorts differ substantially in detection methods, cutoff definitions, patient treatment regimens, and adjustment for clinicopathological confounders, with no independent multicenter validation available to date. For these reasons, H3K18la should be framed as an exploratory, context-dependent marker, instead of a universal biomarker for adverse prognosis.

Mechanistic studies suggest that H3K18la gives tumor cells a proliferative advantage by occupying target-gene promoters and rewiring transcriptional programs. In PDAC models, marked H3K18la enrichment at the promoters of the mitotic checkpoint regulators *TTK* and *BUB1B* drives their transcriptional activation, forming a glycolysis-H3K18la-TTK/BUB1B feed-forward loop that accelerates cell-cycle progression (Li F. et al., 2024). In gastric cancer, H3K18la drives VCAM1 expression and activates AKT-mTOR signaling (Zhao et al., 2024). NLRP12 can also enhance glycolytic flux by preventing ubiquitin-mediated degradation of HK2; the resulting increase in H3K18la initiates *MYC* transcription and closes a metabolic-epigenetic interaction loop (Zhou L. et al., 2025). In HCC, H3K18la can be recognized by the epigenetic reader BRD9, which recruits the ncBAF chromatin-remodeling complex and induces oncogenes such as SPARC and TMEM64 (Wei et al., 2026).

### 3.2 Mechanisms of tumor immune escape

#### 3.2.1 Regulation of immune checkpoint molecules

In NSCLC, H3K18la has been associated with upregulation of POM121, c-Myc nuclear translocation, cooperative occupancy of the *CD274* promoter by POM121/c-Myc, increased PD-L1 expression, and weakened CD8^+^ T-cell cytotoxicity (Zhang et al., 2024a). In HCC, high lactate has been reported to increase KIF20A in an H3K18la-associated axis, whereas H. pylori-related gastric cancer studies implicate the HAS2/c-Myc/PD-L1 pathway (Chen et al., 2026a; Chen et al., 2026b). These disease-specific outputs should not be interpreted as autonomous promoter selection by H3K18la. A testable model is that lactate flux permits deposition, while lineage-specific transcription factors, chromatin accessibility, and recruited cofactors shape locus occupancy; direct factor-occupancy and locus-specific rescue studies are still needed.

#### 3.2.2 Induction of immunosuppressive cell chemotaxis and polarization

Beyond its tumor-cell-intrinsic effects, lactate-driven H3K18la influences stromal and immune-cell fates. In the pancreatic cancer microenvironment, tumor-derived lactate upregulates CXCL1 through PCAF-mediated H3K18la, leading to extensive recruitment of immunosuppressive neutrophils; combined PCAF and PD-1 blockade produces a notable synergistic antitumor effect (Zhang et al., 2026b). Studies in HCC, bladder cancer, cervical cancer, and glioma connect H3K18la to M2 macrophage polarization. In HCC, the splicing factor SRSF10 promotes M2 polarization and reduces CD8^+^ T-cell infiltration through a glycolysis-H3K18la feedback network (Cai et al., 2024). Bladder cancer cells appear to use a related process by regulating PRKN-mediated mitophagy through H3K18la, thereby reinforcing an immunosuppressive microenvironment (Deng et al., 2025). Under hypoxia, lactate can induce M2 macrophage differentiation through H3K18la-mediated GPD2 upregulation in cervical cancer (Huang et al., 2024) and through TNFSF9 upregulation in glioma (Li M. et al., 2024).

#### 3.2.3 Impairment of effector T-Cell cytotoxic function

H3K18la can impair effector immunity through indirect mechanisms. In gastric cancer, it upregulates the RNA methyltransferase METTL3 and enhances m6A-dependent translation of the chaperonin CCT2. Accumulated CCT2 inhibits calcium influx in T cells and promotes CD8^+^ T-cell exhaustion (Qian et al., 2026). In malignant pleural effusion, FOXP3+ NKT-like cells take up lactate through MCT1, which maintains H3K18la-dependent FOXP3 transcription and an immunosuppressive phenotype. Treatment with the MCT1 inhibitor 7ACC2 lowers H3K18la levels and can partly reverse the immune state of “cold” tumors (Wang et al., 2023).

#### 3.2.4 Coordinated metabolic-epigenetic regulation

Within more complex metabolic networks, H3K18la intersects with other epigenetic layers. In infiltrating myeloid cells in colorectal cancer, lactate increases METTL3 expression and directly induces lactylation of the METTL3 zinc-finger domain; this dual mechanism strengthens target RNA binding and activates the JAK1/STAT3 axis through the m6A-YTHDF1 pathway, thereby reshaping myeloid-cell immunosuppression (Xiong et al., 2022). Similarly, in malignant pleural effusion, lactate activates NF-κB-p65 transcription through H3K18la, increases TNFR2 expression, amplifies the suppressive function of regulatory T cells (Tregs), and accelerates disease progression (Xue et al., 2024). Non-coding RNA circuits extend this cross-layer regulation: H19 is linked to a glycolysis-histone-lactylation feedback state in gastric cancer (Tsukihara et al., 2025), whereas circXRN2 restrains a Hippo-H3K18la-LCN2 pathway in bladder cancer (Xie et al., 2023). The evidence supports regulation of H3K18la abundance or downstream signaling, but it does not yet establish direct lncRNA-guided deposition at individual promoters. Accordingly, the pathogenic networks in Figure 2 should be interpreted as integrated metabolic, chromatin, and RNA regulatory systems rather than effects of an isolated histone mark.

### 3.3 Driving tumor invasion and distant metastasis

Epithelial-mesenchymal transition (EMT) and metastasis also depend, at least in part, on H3K18la-mediated transcriptional reprogramming. In breast cancer, H3K18la binds genomic regions associated with *ZWINT, ECT2, ANLN*, and *EZR* and drives genes involved in cell-cycle control and cytoskeletal remodeling, thereby strengthening invasive capacity (Hou et al., 2024). Colorectal liver metastasis (CRLM) appears to use a related mechanism. TROP2 promotes lactate production through the YBX1-HIF-1α pathway and induces H3K18la; H3K18la then activates metastasis-related genes and sustains epigenetic priming at the TROP2 promoter to maintain its expression, forming a self-reinforcing pro-metastatic loop that can be disrupted by HIF-1α inhibitors such as acriflavine can disrupt this cycle (Wang et al., 2026a).

Lactate shuttling within the microenvironment further promotes metastatic behavior. Lactate secreted by pancreatic cancer-associated fibroblasts (CAFs) is taken up by tumor cells, induces H3K18la, and activates neural guidance factors such as L1CAM and SLIT1, providing an epigenetic basis for the prominent perineural invasion (PNI) of pancreatic cancer (Li T. et al., 2025). In clear cell renal cell carcinoma (ccRCC), inactivation of the tumor suppressor VHL induces H3K18la and upregulates PDGFRβ. PDGFRβ signaling then further enhances lactylation, creating a loop that drives invasive progression (Yang et al., 2022). Conversely, endogenous molecules such as *circXRN2* in bladder cancer can inhibit H3K18la and LCN2 expression by activating the Hippo pathway and may therefore exert antimetastatic effects (Xie et al., 2023).

Across the tumor models reviewed here, H3K18la usually participates in feed-forward circuitry rather than acting as an isolated endpoint. Lactate accumulation increases H3K18la at selected loci; the resulting transcriptional changes favor cell-cycle progression, immune evasion, or metastasis and may, in turn, sustain the metabolic state that generated the mark. The most defensible synthesis is therefore circuit based: metabolic substrate availability sets the global opportunity for lactylation, whereas cell-specific recruiters and RNA or protein scaffolds shape locus occupancy, and readers or transcriptional effectors determine the output. Evidence is strongest when all three levels are perturbed independently. Table 1 compares the best-characterized circuits and their potential translation implications.

**TABLE 1:** Representative histone H3 lysine 18 lactylation (H3K18la)-centered circuits implicated in tumor progression.

Tumor context and references	H3K18la-associated circuit	Main cellular effect	Translational angle
Hepatocellular carcinoma (HCC) (Cai et al., 2024; Wei et al., 2026; Chen et al., 2026a)	Glycolysis/SRSF10-H3K18la feedback; BRD9 candidate recognition; KIF20A-c-Myc-PD-L1 signaling	Tumor growth, M2 macrophage polarization, reduced CD8^+^ T-cell infiltration, and immune escape	Potential combination with glycolysis blockade or anti-PD-1 therapy; clinical value remains exploratory
Pancreatic ductal adenocarcinoma (PDAC) (Li et al., 2024a; Li et al., 2025a)	Glycolysis-H3K18la-TTK/BUB1B loop; CAF-derived lactate-H3K18la-L1CAM/SLIT1 axis	Cell-cycle acceleration and perineural invasion	Targeting lactate exchange or CAF-tumor metabolic crosstalk requires validation
Non-small cell lung cancer (NSCLC) (Zhang et al., 2024a)	H3K18la-associated POM121-c-Myc-CD274/PD-L1 axis	PD-L1 upregulation and weakened CD8^+^ T-cell cytotoxicity	Candidate mechanism for immune-checkpoint sensitization; not a validated biomarker
Gastric cancer and H. pylori-associated remodeling (Tsukihara et al., 2025; Zhao et al., 2024; Chen et al., 2026b; Qian et al., 2026)	Low SIRT1/high H3K18la state; H3K18la-VCAM1-AKT-mTOR; HAS2-c-Myc-PD-L1; METTL3-m6A-CCT2 axis	Tumor growth, checkpoint activation, and CD8^+^ T-cell exhaustion	Rationale for combined metabolic, epigenetic, and immune studies
Colorectal cancer and colorectal liver metastasis (Xiong et al., 2022; Wang et al., 2026a)	Lactate-METTL3 lactylation-m6A-YTHDF1-JAK1/STAT3 axis; TROP2-YBX1-HIF-1α-H3K18la loop	Myeloid-cell immunosuppression and liver-metastatic reinforcement	HIF-1α or lactate-pathway disruption may weaken feedback; requires causal validation
Breast cancer (Hou et al., 2024)	H3K18la enrichment at *ZWINT, ECT2, ANLN,* and *EZR*-associated regions	Cell-cycle and cytoskeletal remodeling with increased invasive capacity	Mechanistic association only; not a validated biomarker
Clear cell renal cell carcinoma and bladder cancer (Xie et al., 2023; Yang et al., 2022)	VHL loss-H3K18la-PDGFRβ loop; circXRN2-Hippo-H3K18la-LCN2 inhibitory pathway	Invasion and metastasis, with context-specific endogenous suppression	PDGFRβ/lactylation loops and Hippo-linked suppressors warrant validation
Cervical cancer and glioma (Huang et al., 2024; Li et al., 2024b)	Hypoxia/lactate-H3K18la-dependent upregulation of GPD2 or TNFSF9	M2 macrophage differentiation and immunosuppressive niche formation	Supports targeting hypoxia-lactate signaling in preclinical models

Abbreviations: CAF, cancer-associated fibroblast; ccRCC, clear cell renal cell carcinoma; CRC, colorectal cancer; CRLM, colorectal liver metastasis; *H. pylori*, *Helicobacter pylori*; H3K18la, histone H3 lysine 18 lactylation; HCC, hepatocellular carcinoma; HIF-1α, hypoxia-inducible factor 1α; NSCLC, non-small cell lung cancer; PDAC, pancreatic ductal adenocarcinoma; PD-L1, programmed death-ligand 1.

## 4 Biological effects of H3K18la in inflammatory and immune system diseases

### 4.1 Regulatory roles during inflammatory responses

Inflammation is an innate defense response to infection, tissue injury, or autoimmune stimulation, and its initiation and resolution are accompanied by extensive epigenetic remodeling (Chen et al., 2018). Two zebrafish inflammation models show that H3K18la acts as a metabolism-driven regulator of inflammatory homeostasis. Metformin reduces H3K18la, reactive oxygen species (ROS) generation, and neutrophil recruitment, thereby exerting antioxidant and anti-inflammatory effects (Zhou et al., 2024). By contrast, circadian light disruption activates an H3K18la-ROS positive feedback cascade, amplifies oxidative stress, and worsens neutrophil-mediated injury (Qiu et al., 2024). Sleep deprivation further activates a lactate-H3K18la-retinoic acid receptor-related orphan receptor alpha (RORα) axis in which H3K18la is enriched at the RORα promoter and promotes neutrophil-mediated systemic inflammation (Zhou et al., 2025c).

In sepsis-associated acute kidney injury (SA-AKI), H3K18la deposition at the RhoA promoter enhances RhoA transcription, activating the RhoA/ROCK/Ezrin pathway, increases Ezrin K263 lactylation, and aggravates renal inflammation (Qiao et al., 2024). Lipopolysaccharide stimulation similarly induces macrophage glycolytic reprogramming, lactate accumulation, and increased H3K18la (Zhang et al., 2019). H3K18la is enriched at the promoter of interferon regulatory factor 7 (IRF7) and drives *IRF7* overexpression, a core transcription factor for M1 macrophage polarization; this promotes the release of pro-inflammatory mediators such as TNF-α and IL-1β and aggravates systemic inflammatory injury (Li et al., 2025b). Clinical studies further show that H3K18la is increased in peripheral blood mononuclear cells from patients with septic shock and correlates with infection severity. Mechanistically, H3K18la may participate in compensatory anti-inflammatory responses by regulating cytokine networks and inducing arginase 1 expression (Chu et al., 2022). H3K18la is also increased in bronchoalveolar lavage fluid from patients with sepsis-associated acute respiratory distress syndrome (ARDS), correlates with inflammatory markers and disease-severity scores, and has been proposed as a risk factor for ARDS development that requires further validation (Li et al., 2025c). Together, these studies position H3K18la as an epigenetic node in acute inflammatory cascades.

### 4.2 Driving mechanisms in tissue and organ fibrosis

Persistent chronic inflammation frequently culminates in organ fibrosis, and H3K18la provides a metabolic-epigenetic bridge between inflammatory persistence and tissue remodeling (He et al., 2020). In renal fibrosis, chromatin immunoprecipitation-quantitative polymerase chain reaction (ChIP-qPCR) and related assays show H3K18la enrichment at promoters of profibrotic genes such α-SMA; by altering local chromatin accessibility, H3K18la promotes transcription of these genes and induces excessive synthesis and deposition of extracellular matrix components, including fibronectin and type III collagen (Wang et al., 2026b). H3K18la can also upregulate fibroblast growth factor receptor 4 (FGFR4), worsening kidney injury induced by calcium oxalate crystal deposition (Ye et al., 2025), and can bind the *Rela* promoter to activate NF-κB signaling to convert metabolic reprogramming into a profibrotic phenotype (Chen et al., 2026c). In liver fibrosis models, H3K18la transcriptionally activates ORMDL3 and directly promotes hepatic stellate-cell activation and proliferation (Zhang et al., 2026c). It can also mediate IGFBP7 upregulation, amplifying fibrotic signaling and contributing to cadmium-induced hepatorenal fibrosis (Xie et al., 2025).

In pulmonary disease, increased H3K18la occupancy at the *NOS2* promoter, enhances transcription of this core target of M1 macrophage polarization, promotes a pro-inflammatory macrophage phenotype, and sustains IL-6 and TNF-alpha secretion, thereby accelerating idiopathic pulmonary fibrosis (Shi et al., 2025). H3K18la-mediated upregulation of YTHDF1 also enhances expression of m6A-modified NREP, stimulates abnormal TGF-β1 secretion, and drives fibroblast-to-myofibroblast transition, a central event in arsenic exposure-related pulmonary fibrosis (Wang et al., 2024b). When pulmonary fibroblasts are exposed to combined TGF-β1 and IL-1β stimulation or high exogenous lactate, H3K18la enrichment at profibrotic promoters such as *Acta2* and *Col1a1* increases, further promoting collagen deposition and tissue stiffening (Liu et al., 2024). Thus, H3K18la functions as a metabolic mark that connects inflammatory signaling with profibrotic transcription across the kidney, liver, and lung.

### 4.3 Pathological mediation in autoimmune diseases

In immune-mediated diseases, aberrant H3K18la affects immune-cell differentiation, stromal-cell behavior, and effector functions. In RA, lactate accumulation in the synovial microenvironment increases H3K18la in rheumatoid arthritis synovial fibroblasts (RASFs), conferring excessive proliferative and invasive properties. Mechanistic work indicates that H3K18la activates METTL1 transcription; METTL1 then stabilizes NeuroD1 mRNA through N7-methylguanosine (m7G) modification and upregulates GPX4, allowing RASFs to resist ferroptosis and thereby promoting synovial hyperplasia and joint destruction (Xu et al., 2026). H3K18la enrichment at promoters of genes such as C3AR1 and AKR1C2, involved in chemokine signaling and TRP-channel inflammatory pathways, further amplifies proliferation, invasion, and paracrine inflammation in fibroblast-like synoviocytes (Zheng et al., 2025).

In digestive immune dysregulation, dextran sulfate sodium (DSS)-induced ulcerative colitis models show that lactate accumulation in colonic tissue can trigger H3K18la-mediated transcription of pro-inflammatory genes and worsen mucosal injury (Xu et al., 2024). However, H3K18la may also contribute to immune homeostasis under selected conditions. When exogenous lactate enters macrophages through MCT1, induced H3K18la can reshape macrophage polarization, reduce pro-inflammatory cytokine release, inhibit M1 conversion, and limit excessive NLRP3 inflammasome assembly (Sun et al., 2021). In Sjogren syndrome (SS), excessive H3K18la activates autophagy-related signaling, promotes lymphocyte infiltration into exocrine glands, aggravates xerostomia, and drives disease progression (Chen et al., 2026d). H3K18la has also been proposed as an intervention target in autoimmune uveitis because it links HIF-1α-dependent metabolic reprogramming to neutrophil extracellular trap formation (NETosis) (Liu et al., 2026). These findings suggest that H3K18la-directed modulation may offer a therapeutic entry point for refractory immune and autoimmune diseases, provided that disease context is carefully defined.

These observations do not support a single inflammatory function for H3K18la. Its effect depends on the responding cell population, stimulus, and stage of injury: several acute and autoimmune settings associate the mark with inflammatory or fibrotic transcription, whereas selected macrophage states use it during resolution and restoration of tissue homeostasis. In this proposed model, promoter selection is jointly determined by stimulus-responsive transcription factors and pre-established chromatin states. Meanwhile, the intensity and duration of cellular lactate and lactyl-CoA exposure set the metabolic threshold for this regulatory process. Accordingly, whether the final outcome leans toward tissue repair or pathological progression does not mean H3K18la alone delivers a fixed, on/off regulatory signal.

## 5 Pathophysiological roles of H3K18la in metabolic syndrome and cardiovascular diseases

### 5.1 Epigenetic regulation in diabetes and insulin resistance

In diabetes and its complications, H3K18la operates as a molecular sensor linking metabolic stress to gene expression in insulin-responsive organs and the neuroendocrine system. In diabetes-related central nervous system dysfunction, lactate accumulation in brain parenchyma increases H3K18la in microglia. Increased H3K18la deposition at the *TLR4* promoter activates NF-κB signaling, driving microglial polarization toward a neurotoxic M1 phenotype, and accelerates cognitive impairment (Yang et al., 2025). H3K18la also has context-dependent effects on systemic insulin sensitivity. By regulating the KSRP promoter, it creates a pathological link between osteoblast metabolic imbalance and systemic fibrosis; targeting the H3K18la/KSRP/FSTL1 axis improves insulin resistance and attenuates multi-organ fibrosis (Tang et al., 2026). In diabetic kidney disease (DKD), however, alanyl-tRNA synthetase 1 (AARS1) coordinates lactylation of H3K18 and STAT1, drives ELOVL5 transcription, promotes abnormal polyunsaturated fatty-acid synthesis, and induces ferroptosis in renal tubular epithelial cells, thereby accelerating renal functional decline (Hong et al., 2026). These mechanistic differences underscore the tissue specificity of H3K18la in diabetes and its microvascular complications.

### 5.2 Molecular mechanisms in secondary metabolic comorbidities

H3K18la also participates in secondary or comorbid metabolic disorders. In polycystic ovary syndrome (PCOS), abnormal nuclear translocation of pyruvate kinase M2 (nPKM2) enhances genome-wide H3K18la, remodels local three-dimensional chromatin architecture, and aberrantly activates PCOS susceptibility genes such as *CYP17A1* (Yu et al., 2025). H3K18la therefore acts as an epigenetic imprint of metabolic disturbance and contributes to hyperandrogenism and ovulatory dysfunction (Zhang Y. et al., 2026). In nonalcoholic fatty liver disease (NAFLD) associated with arsenic exposure, mass spectrometry and cleavage under targets and tagmentation (CUT&Tag) analyses indicate that lipid metabolic disturbance depends on H3K18la emergence (Li H. et al., 2025). This mark activates the CD36-NLRP3 inflammasome axis, promotes overexpression of lipid-phagocytic receptors and local inflammation, and becomes a pathogenic route linking environmental exposure to metabolic liver injury (Li H. et al., 2025).

### 5.3 Acute and chronic cardio-cerebrovascular diseases

In aortic dissection, H3K18la contributes to aneurysm or dissection progression by activating p53-mediated apoptotic transcriptional responses in vascular smooth muscle cells (Li et al., 2025e). H3K18la can also activate GATA4 signaling and induce pathological cardiac hypertrophy (Wang et al., 2025a). During pulmonary arterial hypertension, H3K18la upregulates HIF-1α target genes, including *Bmp5*, *Trpc5*, and *Kit*, thereby promoting pulmonary artery smooth muscle cell (PASMC) proliferation and vascular remodeling. Conversely, inhibition of lactate dehydrogenase reduces H3K18la levels and attenuates hypoxic pulmonary hypertension (Chen et al., 2023).

H3K18la also has protective functions during repair after ischemic injury. After myocardial infarction, H3K18la remotely and early activates tissue-repair genes, including *Lrg1*, *VEGF-A*, and *IL-10*, in bone marrow and circulating monocytes, thereby endowing monocyte-macrophages with anti-inflammatory and pro-angiogenic activities and improving the post-infarction repair microenvironment and cardiac function (Wang et al., 2022). H3K18la can also accelerate the transition of cardiac macrophages toward a reparative phenotype and restore immune-inflammatory balance (Su et al., 2025). In ischemic stroke models, increased H3K18la activates *Plxnb2* transcription, promotes microglial polarization toward an anti-inflammatory phenotype, attenuates neuroinflammation, and supports repair of the damaged neurotransmitter system (Li P. et al., 2025).

The contrast between chronic metabolic injury and acute ischemic repair is especially important. Sustained H3K18la in diabetes-associated tissues is commonly linked to maladaptive transcription and organ damage, whereas transient induction after ischemia can activate reparative programs. Duration, cell lineage, and metabolic context therefore need to be reported when these findings are compared. In the absence of dedicated writers or universally validated readers, these opposing effects are better explained by context-specific recruitment and transcription-factor availability than by an intrinsic reparative or pathogenic property of the mark.

## 6 Roles of H3K18la in degenerative diseases

### 6.1 Neurodegenerative diseases

In neurodegenerative disorders, Alzheimer’s disease (AD) models show increased pan-histone lactylation (Pan-Kla) in senescent microglia and hippocampal tissue (Wei et al., 2023). Among these marks, H3K18la is a dominant modification that is highly expressed in the hippocampus (Wei et al., 2023). Elevated H3K18la positively regulates NF-κB signaling and promotes senescence-associated secretory phenotype (SASP) factors such as IL-6 and IL-8, thereby aggravating neuroinflammatory cascades, neuronal senescence, and cognitive decline (Wei et al., 2023).

### 6.2 Musculoskeletal degenerative diseases

Osteoarthritis (OA), the most common degenerative disease of the musculoskeletal system, affects approximately 7% of the global population (Steinmetz et al., 2023). During OA progression, activated fibrochondrocytes use FSTL1 to activate PI3K/mTOR signaling and increase chondrocyte glycolysis, leading to local lactate accumulation and abnormal H3K18la elevation. In the nucleus, H3K18la initiates transcription of profibrotic genes such as *ITGA6, CXCL10*, and *PARP16* and promotes cartilage fibrosis (Lu et al., 2026). In intervertebral disc degeneration, the mitophagy agonist TJ0113 suppresses excessive lactate production in senescent nucleus pulposus cells, reduces H3K18la, blocks *THBS1* transcription and downstream inflammatory amplification, and delays disease progression (Wang et al., 2025b).

By contrast, H3K18la exhibits a bone-protective role in senile osteoporosis. Lactate secreted by glycolytic endothelial cells can act in a paracrine manner on BMSCs and increase in intracellular H3K18la. This epigenetic change activates key osteogenic genes, including *COL1A2, COMP, ENPP1*, and *TCF7L2*, promotes osteoblast differentiation, and alleviates osteoporosis (Wu et al., 2023).

### 6.3 Cardiovascular degenerative diseases

In cardiovascular degenerative disease, H3K18la again shows strong context dependence. MCT4 deficiency enhances p300-mediated H3K18la in macrophages, activating tricarboxylic-acid-cycle-related genes and anti-inflammatory gene clusters. This metabolic-epigenetic reprogramming promotes a tissue-repair macrophage phenotype and reduces atherosclerotic plaque burden (Zhang et al., 2024b). Similarly, Qinggan Jianpi formula (QGJP) inhibits the HIF-1α/MCT4 axis and blocks macrophage lactate efflux. Intracellular lactate accumulation then upregulates H3K18la, activates repair-gene networks, suppresses M1 polarization, and exerts anti-atherosclerotic effects (Zhuang et al., 2026).

Other evidence points to pathogenic effects in vascular degeneration. ASF1A-dependent, p300-mediated H3K18la can aggravate atherosclerosis by driving endothelial-mesenchymal transition (EndMT) through H3K18la enrichment and transcriptional activation at the *SNAI1* promoter. Targeted glycolysis inhibition or HK2 degradation reduces H3K18la and attenuates vascular intimal lesions (Lv et al., 2026). Narciclasine likewise inhibits GLUT1-and PFKFB3-mediated glycolysis, reduces H3K18la, blocks abnormal NF-κB activation, improves endothelial inflammation, and restrains early atherosclerosis (Wang et al., 2026c). In vascular aging, however, TRPM7 maintains moderate endothelial lactate production and p300-mediated H3K18la, suppresses the senescence marker p21, and promotes pro-angiogenic gene transcription, thereby delaying endothelial senescence (Wang et al., 2025c).

Degenerative-disease studies likewise indicate that the direction of effect is conditional. Persistent H3K18la accompanies inflammatory and fibrotic deterioration in senescent neural, articular, and disc tissues, whereas controlled induction in bone or selected vascular cells supports osteogenesis, reparative macrophage states, or endothelial resilience. This pattern supports a permissive-cofactor model in which H3K18la amplifies transcriptional programs selected by cell identity and injury signals, rather than functioning as a self-sufficient determinant of biological direction.

## 7 Translational challenges and future directions

The evidence summarized above makes H3K18la a plausible translational candidate, but not yet a clinically mature one (Figure 2). Lactate availability, chromatin state, and disease-relevant transcription are experimentally connected; however, the direction and magnitude of the response vary with tissue, disease stage, cell lineage, and local metabolism. These variables must be built into biomarker and intervention studies rather than treated as sources of residual heterogeneity. The major translational challenges and future research priorities are summarized in Table 2.

**TABLE 2:** Translational challenges and future priorities for H3K18la-centered metabolic-epigenetic circuits.

Translational context and references	H3K18la-related obstacle	Clinical implication	Research priority
Biomarker development (Hou et al., 2025; Chu et al., 2022; Li et al., 2025c)	Human cohorts are limited, mostly single-center, and use heterogeneous assays and cutoffs	Associations support investigation but do not establish diagnostic, prognostic, or predictive utility	Use prospective multicenter cohorts, prespecified thresholds, external validation, and incremental-value analyses
Therapeutic targeting (Kaczmarska et al., 2017; Huang et al., 2018; Sabari et al., 2015; Chen et al., 2022; Lasko et al., 2017; Yang et al., 2026)	C646 and related p300/CBP perturbations alter H3K18ac, other acyl marks, and non-histone substrates	Drug effects cannot be attributed exclusively to H3K18la without mark-specific target engagement and rescue	Pair inhibitors with H3K18ac/pan-acylation controls, orthogonal perturbations, and locus-specific rescue
Detection standardization (Zhang et al., 2019; Moreno-Yruela et al., 2022; Zhu et al., 2025)	Antibody performance, enrichment methods, and MS workflows vary substantially	H3K18la abundance and genomic localization are difficult to compare across studies	Use validated antibodies, reference materials, orthogonal assays, and unified pipelines
Context-dependent intervention (Wang et al., 2022; Wang et al., 2025b; Bao et al., 2025)	Pathogenic roles in tumors coexist with repair-promoting roles in ischemia and bone formation	Uniform inhibition or activation may block protective programs or miss disease drivers	Stratify by disease stage, cell type, target locus, and metabolic microenvironment
Mechanistic prioritization (Wang et al., 2024a; Zhu et al., 2025; Dong et al., 2024; Tsai et al., 2010; Tsukihara et al., 2025; Xie et al., 2023; Wei et al., 2026; Flynn et al., 2015; Li et al., 2016; Zhang et al., 2016)	No dedicated writer/eraser or universally validated reader explains locus selection; direct lncRNA tethering remains unproven	H3K18la is best treated as a context-dependent permissive mark embedded in protein, RNA, and transcription-factor networks	Test recruiter and reader binding, RNA scaffolds, chromatin accessibility, and H3K18la/H3K18ac causality in the same model

Abbreviations: CRISPR-dCas9, clustered regularly interspaced short palindromic repeats-deactivated Cas9; H3K18la, histone H3 lysine 18 lactylation; MS, mass spectrometry.

### 7.1 H3K18la as a candidate biomarker

Biomarker claims should be grounded in human cohort evidence and should be separated from mechanistic or preclinical findings. Available clinical studies include peripheral-blood mononuclear-cell measurements in septic shock (Chu et al., 2022), bronchoalveolar-lavage-fluid measurements in sepsis-associated acute respiratory distress syndrome (Li X. et al., 2025), and a pancreatic-cancer cohort relating circulating or tissue H3K18la to disease severity (Hou et al., 2025). Some studies involving patient-derived tumor tissues place H3K18la within prognostically relevant SRSF10-, KRT19-, H19-, or glycolysis-centered circuits (Cai et al., 2024; Tsukihara et al., 2025; Li et al., 2024a; Zhang et al., 2026a), but these studies do not independently establish H3K18la as a diagnostic, prognostic, or predictive biomarker. Overall, the available evidence supports disease-associated correlations rather than clinical utility: most studies are single-center, retrospective or exploratory, use non-harmonized assays and cutoffs, and lack external validation, prespecified thresholds, calibration, or evidence that H3K18la adds value beyond established clinical variables. Cell-line and animal findings can motivate mechanistic hypotheses but should not be cited as biomarker validation. Future studies require prospective multicenter cohorts, blinded assay assessment, predefined endpoints, comparison with H3K18ac and pan-lactylation, and reporting of discrimination, calibration, reproducibility, and incremental clinical benefit before H3K18la is used for diagnosis, prognosis, or treatment selection.

### 7.2 Therapeutic targeting of lactate-H3K18la circuits

Therapeutic studies using p300/CBP inhibitors, lactate-modulating agents, or genetic depletion provide pathway-level proof of concept but do not yet validate H3K18la as the therapeutic target (Yang et al., 2026). C646 and related p300/CBP inhibitors are not modification specific (Chen et al., 2022). Biochemical and structural work shows that p300/CBP catalysis spans multiple acyl marks (Kaczmarska et al., 2017; Huang et al., 2018; Sabari et al., 2015), so inhibitor phenotypes may reflect transcriptional changes independent of H3K18la. Consequently, drug response should not be attributed exclusively to H3K18la unless the study demonstrates target engagement at H3K18, excludes H3K18ac and pan-acylation as alternative mediators, and rescues the phenotype by restoring H3K18la at the relevant locus. Even A-485, despite improved catalytic selectivity for p300/CBP, remains modification nonspecific (Lasko et al., 2017). Next-generation strategies should prioritize disruption of disease-specific writer-recruiter complexes, metabolic control of lactyl-CoA, or clustered regularly interspaced short palindromic repeats-deactivated Cas9 (CRISPR-dCas9)-based locus- and time-specific editing, with parallel monitoring of protective H3K18la programs in ischemic and reparative tissues.

### 7.3 Methodological limitations in H3K18la detection

A major translational bottleneck is methodological inconsistency. Current detection strategies vary across laboratories in antibody specificity, antibody affinity, sample preparation, chromatin enrichment, and mass-spectrometry quantification. These variables can strongly affect apparent H3K18la abundance and genomic localization, making cross-study comparison difficult. Standardized quantitative systems are still lacking, especially for distinguishing H3K18la from global histone lactylation or neighboring H3 modifications. Progress will require validated antibodies, orthogonal confirmation by mass spectrometry or chromatin-based assays, shared reference materials, transparent reporting of experimental conditions, and harmonized analytical pipelines. To support causal inference, ChIP enrichment assays should be combined with perturbation of the putative recruiter or reader. Proper controls including H3K18ac, total H3, chromatin accessibility and transcription factor occupancy must be included for parallel comparison. Such experiments help differentiate locus-specific H3K18la regulatory mechanisms from passive buildup of this modification at pre-activated chromatin regions.

### 7.4 Context-dependent risk of H3K18la intervention

H3K18la exerts markedly distinct biological functions depending on the tissue microenvironment, exhibiting prominent functional duality. In multiple solid tumors, lactate accumulation has been associated with elevated H3K18la levels (Figure 2). In the reported models, this modification is associated with oncogenic transcription programs, facilitates tumor immune escape, and enhances cancer invasion and metastasis (Bao et al., 2025). In contrast, H3K18la plays a protective role under cardiac and cerebral ischemia–reperfusion conditions, where it triggers antioxidant, anti-apoptotic, and tissue repair responses to alleviate stress injury (Wang et al., 2022). A similar protective phenotype is observed during osteoblast differentiation; H3K18la promotes bone regeneration and may serve as a beneficial epigenetic modification against degenerative bone loss (Wu et al., 2023).

Such context-dependent functions suggest that H3K18la acts primarily as a permissive epigenetic mark embedded in diverse regulatory networks. Metabolic lactate signaling governs H3K18la deposition, whereas the ultimate biological outcome—either tissue repair or pathological progression—is dictated by local transcription factor activity, chromatin accessibility, protein or RNA scaffolds, and downstream effector pathways. Accordingly, H3K18la cannot be targeted through a universal therapeutic strategy. Future interventions should be tailored to specific disease types, pathological stages, cell lineages, genomic loci, local metabolic status, and the competing regulatory effects of H3K18ac.

### 7.5 Future priorities

Progress now depends on six linked tasks: defining the enzymes, readers, precursors, transcription factors, and protein or RNA scaffolds that regulate specific loci; biochemically testing candidate readers against H3K18la and competing acyl-lysine marks; separating causal H3K18la programs from correlated responses to lactate or H3K18ac; harmonizing assays across laboratories; testing circuit-selective rather than global interventions; and validating safety and clinical performance in well-annotated human cohorts. Priority experiments should integrate metabolite measurements, H3K18la/H3K18ac chromatin maps, RNA-scaffold mapping, reader occupancy, chromatin accessibility, and locus-specific rescue in the same model. Integration with established metabolic, inflammatory, and molecular markers will be more informative than evaluating H3K18la in isolation.

## 8 Conclusion

H3K18la provides a mechanistic route through which metabolic change can influence chromatin and transcription. The studies reviewed here connect this route to tumor growth, immune-cell state, fibrosis, metabolic injury, vascular responses, degeneration, and tissue repair. They also show why total lactate or pan-lactylation cannot substitute automatically for locus- and cell-specific measurement of H3K18la.

Clinical use remains premature because the relevant writers, erasers, readers, causal loci, and assay standards are not sufficiently defined, and because pathogenic and reparative effects can coexist. A two-stage framework reconciles these observations: lactate and lactyl-CoA create metabolic permission for H3K18la, whereas cell-specific transcription factors, chromatin accessibility, and protein or RNA scaffolds provide locus selection; candidate readers and transcriptional effectors then amplify the local program. This framework remains a testable model rather than an established pathway (Figure 3). The next stage of the field should therefore emphasize standardized measurement, H3K18ac-aware and inhibitor-independent tests of causality, structural validation of readers, and prospective validation in human cohorts. Such evidence will determine whether H3K18la becomes a useful component of biomarker panels or a tractable target for context-specific intervention.

**FIGURE 3:**
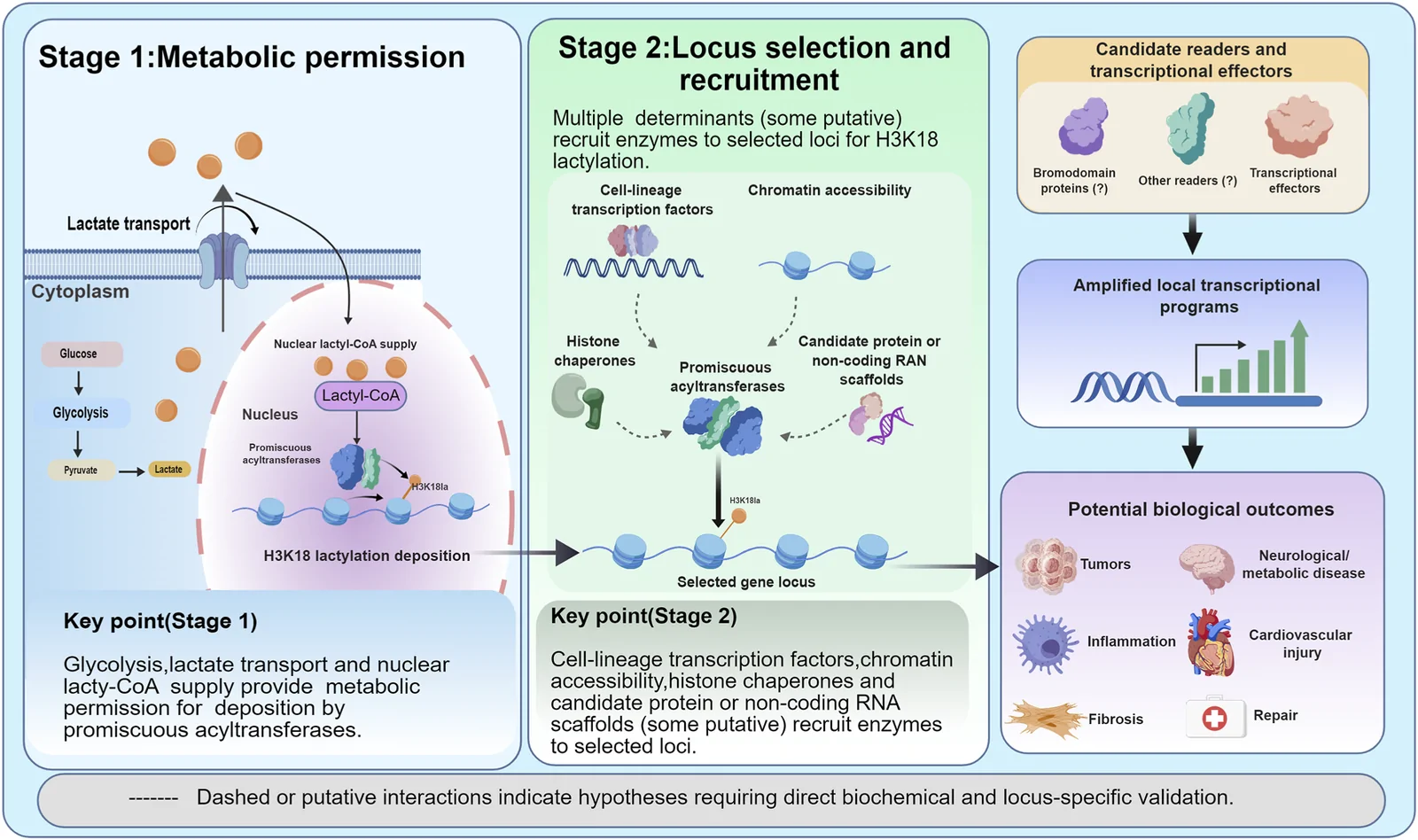
Proposed two-stage model of H3K18 lactylation as a metabolic-epigenetic hub. Glycolysis, lactate transport, and nuclear lactyl-CoA supply provide metabolic permission for deposition by promiscuous acyltransferases. Cell-lineage transcription factors, chromatin accessibility, histone chaperones, and candidate protein or non-coding RNA scaffolds are proposed to recruit these enzymes to selected loci. Candidate readers and transcriptional effectors then amplify local programs governing tumors, inflammation, fibrosis, neurological/metabolic disease, cardiovascular injury, or repair. Dashed or putative interactions should be interpreted as hypotheses requiring direct biochemical and locus-specific validation.

## Glossary

7ACC2: 7-amino-2-chloro-4-hydroxy-quinoline-3-carbonitrile (MCT1 inhibitor)
A-485: Potent and selective catalytic inhibitor of p300/CBP
AD: Alzheimer’s disease
ARDS: Acute respiratory distress syndrome
BioGDP: Generic Diagramming Platform (https://biogdp.com)
BMSC: Bone marrow mesenchymal stem cell
C646: Competitive pyrazolone inhibitor of p300/CBP catalytic activity
CA19-9: Carbohydrate antigen 19–9
CAF: Cancer-associated fibroblast
ccRCC: Clear cell renal cell carcinoma
CEA: Carcinoembryonic antigen
ChIP: Chromatin immunoprecipitation
ChIP-qPCR: Chromatin immunoprecipitation-quantitative polymerase chain reaction
ChIP-seq: Chromatin immunoprecipitation sequencing
ChIRP: Chromatin isolation by RNA purification
ChIRP-seq: Chromatin isolation by RNA purification sequencing
circRNA: Circular RNA
CLIP: Cross-linking and immunoprecipitation
CRC: Colorectal cancer
CRISPR-dCas9: Clustered regularly interspaced short palindromic repeats-deactivated Cas9
CRLM: Colorectal liver metastasis
CUT&Tag: Cleavage under targets and tagmentation
DKD: Diabetic kidney disease
DSS: Dextran sulfate sodium
EMT: Epithelial-mesenchymal transition
EndMT: Endothelial-mesenchymal transition
H. pylori: *Helicobacter pylori*
H3K18ac: Histone H3 lysine 18 acetylation
H3K18la: Histone H3 lysine 18 lactylation
HCC: Hepatocellular carcinoma
lncRNA: Long non-coding RNA
m6A: N6-methyladenosine
m7G: N7-methylguanosine
MS: Mass spectrometry
NAFLD: Nonalcoholic fatty liver disease
ncBAF: Non-canonical BAF chromatin remodeling complex
NETosis: Neutrophil extracellular trap formation
NKT: Natural killer T cell
NSCLC: Non-small cell lung cancer
OA: Osteoarthritis
Pan-Kla: Pan-histone lysine lactylation
PASMC: Pulmonary artery smooth muscle cell
PCOS: Polycystic ovary syndrome
PD-1: Programmed cell death protein 1
PD-L1: Programmed death-ligand 1
PDAC: Pancreatic ductal adenocarcinoma
PNI: Perineural invasion
PTM: Post-translational modification
QGJP: Qinggan Jianpi formula
RA: Rheumatoid arthritis
RASF: Rheumatoid arthritis synovial fibroblast
RNA-seq: RNA sequencing
ROS: Reactive oxygen species
SA-AKI: Sepsis-associated acute kidney injury
SASP: Senescence-associated secretory phenotype
SS: Sjögren syndrome
TIME: Tumor immune microenvironment
Tregs: Regulatory T cells
